# Plasmonic slanted slit gratings for efficient through-substrate light-plasmon coupling and sensing

**DOI:** 10.1038/s41598-024-52564-9

**Published:** 2024-01-24

**Authors:** Fatemeh Fouladi Mahani, Luis Angel Mayoral Astorga, Hyung Woo Choi, Arash Mokhtari, Pierre Berini

**Affiliations:** 1 Optical and RF Communication Systems (ORCS) Lab, Electrical Engineering Department, Shahid Bahonar University of Kerman, Kerman, 7616913439 Iran; 2 School of Electrical Engineering and Computer Science, University of Ottawa, Ottawa, ON K1N 6N5 Canada; 3 School of Electrical Engineering and Computer Science, Department of Physics, University of Ottawa, Ottawa, ON K1N 6N5 Canada

**Keywords:** Optics and photonics, Applied optics, Optical sensors

## Abstract

We present an experimental study of plasmonic slanted slit gratings (PSSGs) designed to achieve directional coupling between an incident light beam and surface plasmon polaritons (SPPs) propagating along the surface of the structure. We also investigate mirrored PSSG pairs interconnected by a plasmonic slab waveguide. The structures are fabricated using direct milling by a gallium focused ion beam (FIB). In a mirrored pair arrangement, the first PSSG couples a perpendicularly-incident light beam to SPPs propagating in one direction along the waveguide, while the second PSSG decouples SPPs to perpendicularly-emerging light. This configuration shows promise for sensing applications due to the high sensitivity of the excited SPPs to changes in the refractive index of the bounding medium, and the separation of the optics from the fluidics by the substrate. The design also exhibits robustness to fabrication tolerances. The optical characteristics and sensing potential are investigated theoretically and experimentally, highlighting its potential for a wide range of applications.

## Introduction

Surface plasmon polaritons (SPPs) enhance light-matter interactions at the nanoscale, leading to numerous applications in sensing, imaging, and optical communications^1,2^. Efficient directional coupling between light beams and SPPs is a fundamental challenge in enabling advanced optical functionalities and applications, *e.g.,* integrated nanophotonic circuits and sensing.

Various structures and techniques have been explored to address the challenge of efficient light-SPP coupling. These include grooves^3^, slits^4–6^, slit-grooves^7,8^, Bragg mirrors^9^, ridge (grating) couplers^10^, and nano-antennas^11^, each offering unique coupling mechanisms and profiles with inherent advantages and limitations. For instance, single slits and grooves provide strong field confinement, but their coupling efficiency is limited by the small slit size, resulting in high coupling losses. Grating couplers with asymmetric profiles have shown promise for top-side illumination^12–15^. However, their suitability for perpendicular coupling through the metal film, which is desirable, *e.g.*, in biosensor configurations where the optics and the fluidics are on opposite sides of the substrate, remains a challenge. This limitation underscores the need for novel structures that can enable efficient light-SPP coupling perpendicularly through the metal film, and preferentially exciting SPPs along one propagation direction.

This study presents the fabrication and characterization of plasmonic slanted slit gratings (PSSGs), designed to couple a perpendicularly-incident light beam to SPPs propagating unidirectionally. A notable departure from predominantly discussed top-illuminated configurations in the literature is our introduction of a through-substrate coupling approach. This approach significantly enhances the versatility of a setup by allowing the spatial separation of fluidics and optics, providing tangible advantages for sensing and biosensing applications. Moreover, we introduce the novel concept of mirrored plasmonic PSSG pairs interconnected by a plasmonic slab waveguide, which facilitates transmission-based interrogation and is advantageous in sensing applications. Beyond theoretical considerations, our study reports the fabrication and experimental characterization of PSSGs, offering a realistic perspective through experimental exploration. We also demonstrate the potential of the proposed structure for sensing applications, offering a good practical sensitivity measured through a robust interrogation setup. This approach underscores the reliability and practical applicability of our design in real-world scenarios.

## Structure

**Figure 1:**
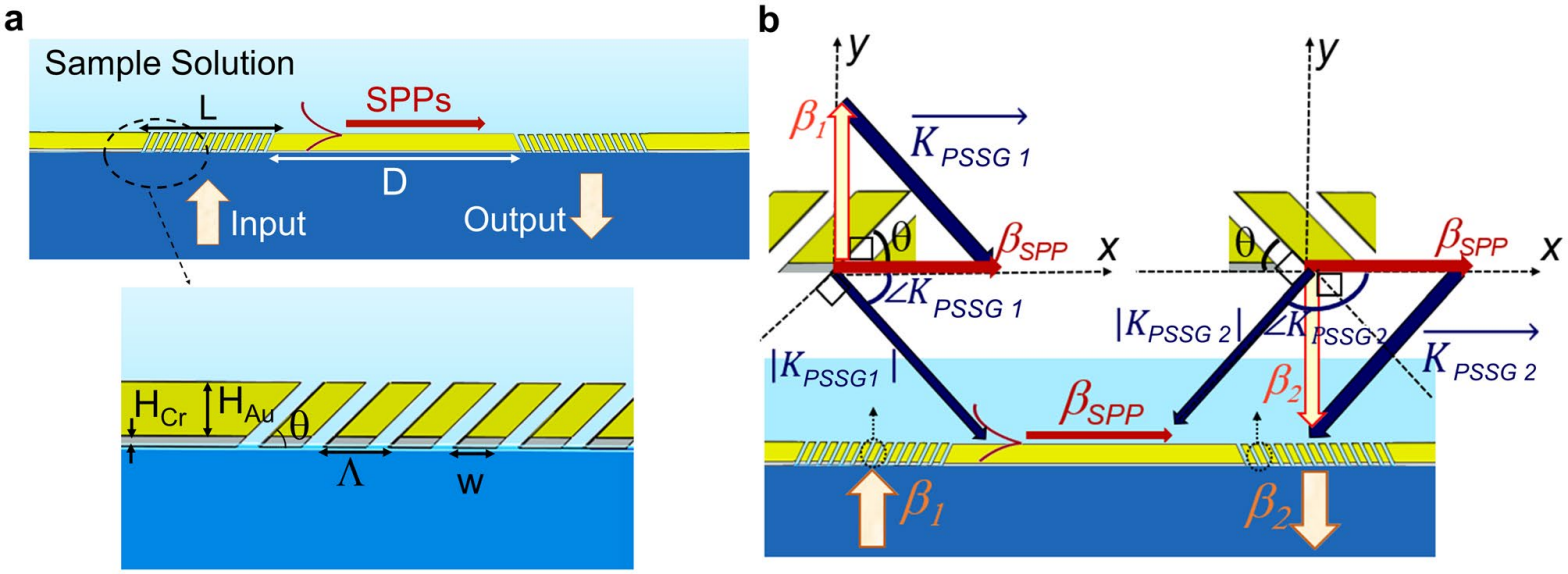
Schematic illustration of the proposed structure based on plasmonic slanted slit gratings (PSSGs). (**a**) 2D overview of the structure showcasing the arrangement of PSSGs along with a zoomed-in cross-sectional sketch of one of the PSSGs. (**b**) Vector geometry used for the theoretical design of the PSSGs.

The Fig. 1a presents a cross-sectional view of the proposed structure, featuring mirrored Au/Cr PSSGs interconnected by an Au/Cr waveguide on a glass substrate. Transverse magnetic (TM)-polarized light interacts with the structure through the substrate, emerging at the first PSSG, where it couples to SPPs propagating along the top surface of the plasmonic waveguide. The second PSSG redirects the SPPs, allowing the outcoupled light to emerge perpendicularly for detection. The SPPs propagating along the plasmonic waveguide are highly sensitive to changes in the bounding medium's refractive index, highlighting the structure's potential for sensing applications.

In our theoretical design, we assume an Au layer with a thickness of 300 nm, on a 30 nm thick Cr layer. As will be discussed later, selecting a thick Au layer facilitates efficient light-SPP coupling owing to the high refractive index contrast of the PSSGs. Au also offers good chemical stability and ease of functionalization, making it well-suited for practical sensing and biosensing applications. The relatively thick Cr layer effectively suppresses the undesired excitation of SPPs at the bottom interface^16^, since the bottom SPPs do not contribute to sensing. The optical constants of Au and Cr used in the simulations were obtained from experimental data^17,18^.

In order to analyze the behavior of the device and gain insight into the excited modes, we conducted finite element method (FEM) simulations utilizing boundary mode analysis (BMA) in a simplified 2D model to make the design more straightforward^19^. A slab waveguide with appropriate core and cladding characteristics was used to model an input Gaussian beam emerging from a single-mode optical fiber. The system was configured with input and output ports, and BMA steps were performed at these ports, accompanied by a comprehensive frequency response analysis of the entire structure. To ensure accurate and reliable results, our electromagnetic computations incorporated perfectly matched layers (PMLs) and scattering boundary conditions, effectively minimizing unwanted reflections from the boundaries of the simulation domain.

The targeted operating free-space wavelength of the structure is ${\lambda }_{0}=1.31 \mathrm{\mu m}$, as high-quality and cost-effective optoelectronics are available near this wavelength. In order to achieve efficient PSSGs with a compact design for directional coupling, a strong index modulation is needed^20^. To fulfill this requirement, we employ Au/Cr PSSGs with parallelogram-shaped ridges.

To design the PSSGs for efficient directional coupling, we utilize the same procedure as outlined in our previous studies^21,22^. The design aims to couple the input TM_0_ mode (propagation constant ${\beta }_{1}$) to SPPs propagating along the top of the plasmonic waveguide (propagation constant ${\beta }_{SPP}$), followed by outcoupling of the SPPs to the output TM_0_ mode (propagation constant ${\beta }_{2}$). A schematic representation of the signal path and design steps can be seen in Fig. 1b.

The simplified vector diagram in Fig. 1b illustrates that the wavevector of the first PSSG, denoted ${K}_{PSSG 1}$, can be approximated as the vector difference between ${\beta }_{1}$ and ${\beta }_{SPP}$, which are obtained from modal computations. By leveraging these geometric equations, we can estimate the PSSG wavevector ${(K}_{PSSG})$, propagation constant (${\beta }_{PSSG}$), slant angle ($\theta$), and period ($\Lambda$), as presented in Table 1:

1 $$ \left| {K_{PSSG} } \right|_{ } = \sqrt {\left| {\beta_{1} } \right|^{2} + \left| {\beta_{SPP} } \right|^{2} } $$

2 $$ \theta = \sin^{ - 1} \left( {\frac{{\beta_{SPP} }}{{\left| {K_{PSSG} } \right|}}} \right) $$

3 $$ \beta_{PSSG} = K_{PSSG} \sin (\theta ) $$

4 $$ \Lambda_{ } = \frac{2\pi }{{\beta_{PSSG} }} $$

5 $$ n_{PSSG} = \frac{{\beta_{PSSG} }}{{k_{0} }} $$

**Table 1:** Theoretical design specifications of the PSSG configuration.

Parameter	Definition	Parameter value
$\theta$	Slant angle	45^o^
$\Lambda$	Grating pitch	1307 nm (air-optimized PSSG) 980 nm (water-optimized PSSG)
${\text{ff}}$	Fill factor	60%
${\text{W}}$	Grating width	784 nm (air-optimized PSSG) 588 nm (water-optimized PSSG)
${{\text{H}}}_{{\text{Cr}}}$	Chromium height	30 nm
${{\text{H}}}_{{\text{Au}}}$	Gold height	300 nm
${\text{D}}$	Distance between the gratings	40 μm
${\text{L}}$	Grating length	16 μm

The free-space wavenumber is ${k}_{0}=2\pi /{\lambda }_{0}$. Two PSSG were designed, one optimized for efficient operation in air, while the other is tailored for operation in aqueous environments. The value of $\Lambda$ for each design was scaled to align the resonance wavelength at $1.31 \mathrm{\mu m}$, as shown in Table 1.

To achieve efficient directional coupling, we set the effective index of the PSSGs, ${n}_{PSSG}$, equal to the real part of the effective index of the SPPs, ${n}_{SPP}$, (*i.e*., ${\beta }_{PSSG}= {\beta }_{SPP}$). Using the periodicity and slant angle, we determined the effective index of the PSSG to ensure the creation of a physical SPP mode with significant field enhancement along the top side of the slanted ridges. We manipulated the fill factor ($\mathrm{ff }=\mathrm{ w}/\Lambda$) to match the real part of ${n}_{PSSG}$ to that of ${n}_{SPP}$. The obtained design parameters of the PSSGs are summarized in Table 1.

We selected the distance between the two PSSGs, denoted as ${\text{D}}$, to be approximately $40 \mathrm{\mu m}$. This distance ensures a well-suited separation between the input and output gratings while remaining within the propagation length of the SPPs.

Moreover, we set the length of the gratings, denoted as ${\text{L}}$, to be $16 \mathrm{\mu m}$. This length was selected to ensure that sufficient grating ridges overlap with the incident beam, optimizing the desired directional coupling efficiency. To verify the design, extensive 2D FEM-based simulations were conducted to validate the entire system's performance. The results will be discussed in the following section.

### Modelling results

**Figure 2:**
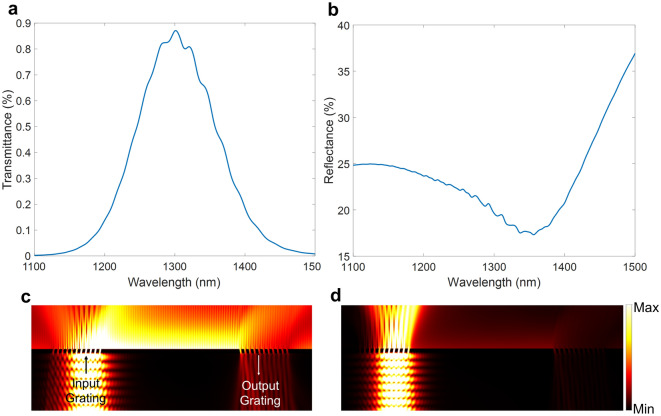
Simulated behavior of the air-optimized PSSG structure. Simulated (**a**) transmittance and (**b**) reflectance spectra of the air-optimized PSSG structure. (**c**) Normalized electric field distribution of the air-optimized PSSG structure on-resonance, indicating the coupling and decoupling of light to SPPs propagating between the two PSSGs. (**d**) Electric field distribution of the air-optimized PSSG structure off-resonance.

The Fig. 2 illustrates the simulated behavior of the air-optimized PSSG structure. In our analysis, we calculate the transmittance from the TM_0_ mode incident on the first PSSG to the TM_0_ mode outcoupled by the second PSSG. This metric effectively encapsulates the overall performance of the structure, including the coupling efficiency of both gratings and SPP propagation along the slab waveguide section. The transmittance was calculated by computing overlap integrals, specifically, the overlap integral between the fields emerging from the output grating and the output TM_0_ mode, when the input grating was excited by the TM_0_ mode. The reflectance was computed as the power reflected back from the first PSSG in the TM_0_ mode, also using overlap integrals.

The transmittance, which therefore represents the ratio of power transmitted into the output TM_0_ mode to the power carried by the input TM_0_ mode, is illustrated in Fig. 2a. Despite the large separation between the two gratings, the structure achieves a reasonable transmittance, sufficient for detection purposes, highlighting the effectiveness of our design approach for PSSGs. Clear evidence of directional coupling is apparent from the on-resonance electric field profile displayed in Fig. 2c, particularly when contrasted with the off-resonance electric field profile presented in Fig. 2d. It is noted that the interaction of light with the slanted grating also results in the excitation of SPPs propagating toward the left of the input grating, so coupling is not strictly one-sided, however, the undesired left-directed coupling is very weak compared to coupling toward the right.

In addition, the reflectance of the input beam from the first PSSG is also extracted as shown in Fig. 2b. The reflectance curve exhibits a resonance behavior over the coupling bandwidth of the structure. In the measurements, we will use this reflectance response to validate and assess the alignment and efficiency of our experimental setup. The non-coincidence of the transmittance peak and reflectance dip in wavelength observed in Fig. 2a,b arises because the transmittance represents the power transmitted into the output TM_0_ mode to the power carried by the input TM_0_ mode, whereas the reflectance corresponds to the power reflected into the TM_0_ mode by the input PSSG. Thus, the former includes coupling from the TM_0_ mode to SPPs by the input PSSG, followed by propagation of SPPs, and outcoupling of SPPs into the output TM_0_ mode by the output PSSG. This produces a different spectral response compared to the reflectance from the input PSSG only.

An attractive characteristic of this structure is its robustness to small variations in the design parameters, thereby ensuring fabrication tolerance. Supplementary Figures S1 and S2 provide more detailed information on design robustness, which is of paramount importance for practical applications.

## Experimental results

### Fabricated structure

Focused ion beam (FIB) milling was employed to fabricate ordered arrays of slanted slits forming PSSG structures (fabrication details are given under the “Fabrication Methodology” section of the Supplementary Information file).

**Figure 3:**
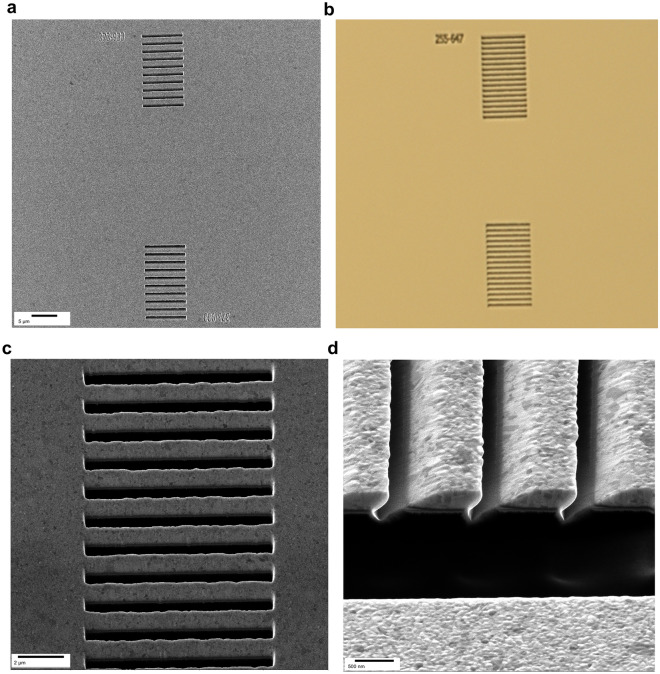
Microscope images of the PSSG structures. (**a**) Helium ion microscope image and (**b**) optical microscope image of the fabricated PSSG structures illustrating the top-view overall layout for the air-optimized and water-optimized PSSGs, respectively. (**c**) Closer view of one PSSG captured with helium ion microscopy. (**d**) Cross-sectional helium ion microscope image of one PSSG structure, providing details of the realized slanted gratings.

The Fig. 3a,b gives top views of fabricated PSSGs optimized for operation in air and in water, respectively. A magnified view of one PSSG obtained by helium ion microscopy is also given in Fig. 3c. Figure 3d shows a cross-sectional view, revealing the slight asymmetric shape of the milled trenches. The fabricated PSSGs have a fill factor of 60% with an error of 1.6%, and the realized slant angle varies from 40° to 54°. The asymmetric trenches are attributed to a slight difference in the working distance originating from the FIB source, which was angled at 46° with respect to the surface of the Au film. As shown in Fig. 3a,b, the length of the PSSGs is 15 ± 1 µm from the first trench to the last one, which produces a variation in the working distance of ~ 200 µm, causing slight defocusing of the ion beam and consequently varying slit angles. Despite these process variations, the PSSGs demonstrate considerable uniformity to minor dimensional discrepancies of less than 14° per trench, and to divergence between the intended and actual patterns as displayed in Supplementary Figure S1. The structure exhibits good robustness against small errors, such as non-parallel side facets of the trenches and differences in depth profile resulting from the angled milling process, which leads to partial removal (< 100 nm) of the quartz substrate (see Supplementary Figure S1 for more details).

### Experimental setup and characterization method

**Figure 4:**
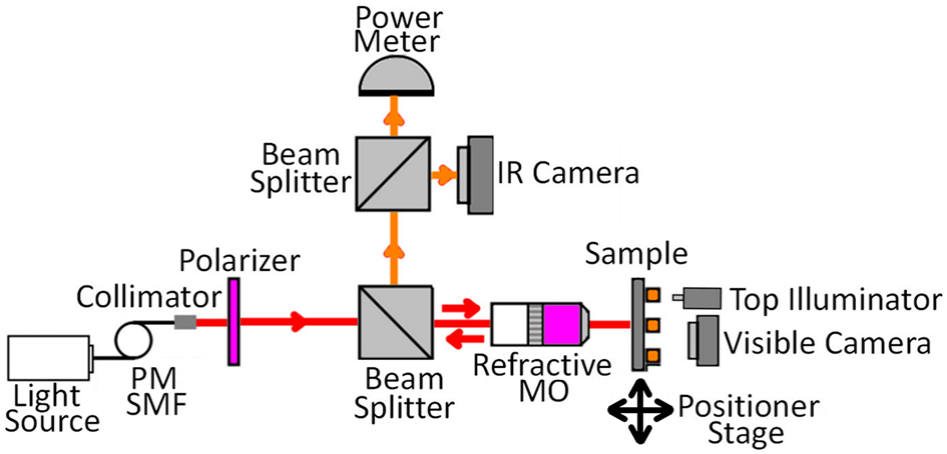
Sketch of the experimental setup used for measuring the response of the PSSG structure.

The Fig. 4 presents a sketch of the experimental setup utilized to measure the optical characteristics of the proposed structure. We employed two light sources to validate our measurement results: a non-coherent supercontinuum light source (NKT Photonics, SuperK Extreme) and a tunable laser (Agilent 8164A) operating from 1260 to 1380 nm.

The input light was delivered using a polarization maintaining single-mode optical fiber. A linear polarizer (LPNIR100) was incorporated to ensure the electric field's orientation was perpendicular to the grating trenches. The beam was directed to a beam splitter (BSW29), and 50% was directed into a plan apochromatic NIR objective (MO, R18091161 50X, NA = 0.42) to focus the incident beam onto the device through the substrate.

The light emerging from the sample was directed through the same beam splitter to the output arm of the set-up. The output arm contains another beam splitter, directing some of the light towards an infrared camera (MicronViewer 7290A) equipped with a frame grabber card (Ophir-Spiricon). This arrangement helps extract and visualize images from the device. Additionally, as required, a top illuminator was used to project an image of the gratings on the IR camera. The remaining beam fraction was directed into a power meter head (InGaAs 81525A) to measure the optical power. The light emerging from the sample has two components: light reflected by the input grating, and light outcoupled by the output grating due to the excitation and propagation of SPPs on the surface of the sample (*cf*. Figures 1a and 2c). The set-up was designed to distinguish between these two components by imaging.

**Figure 5:**
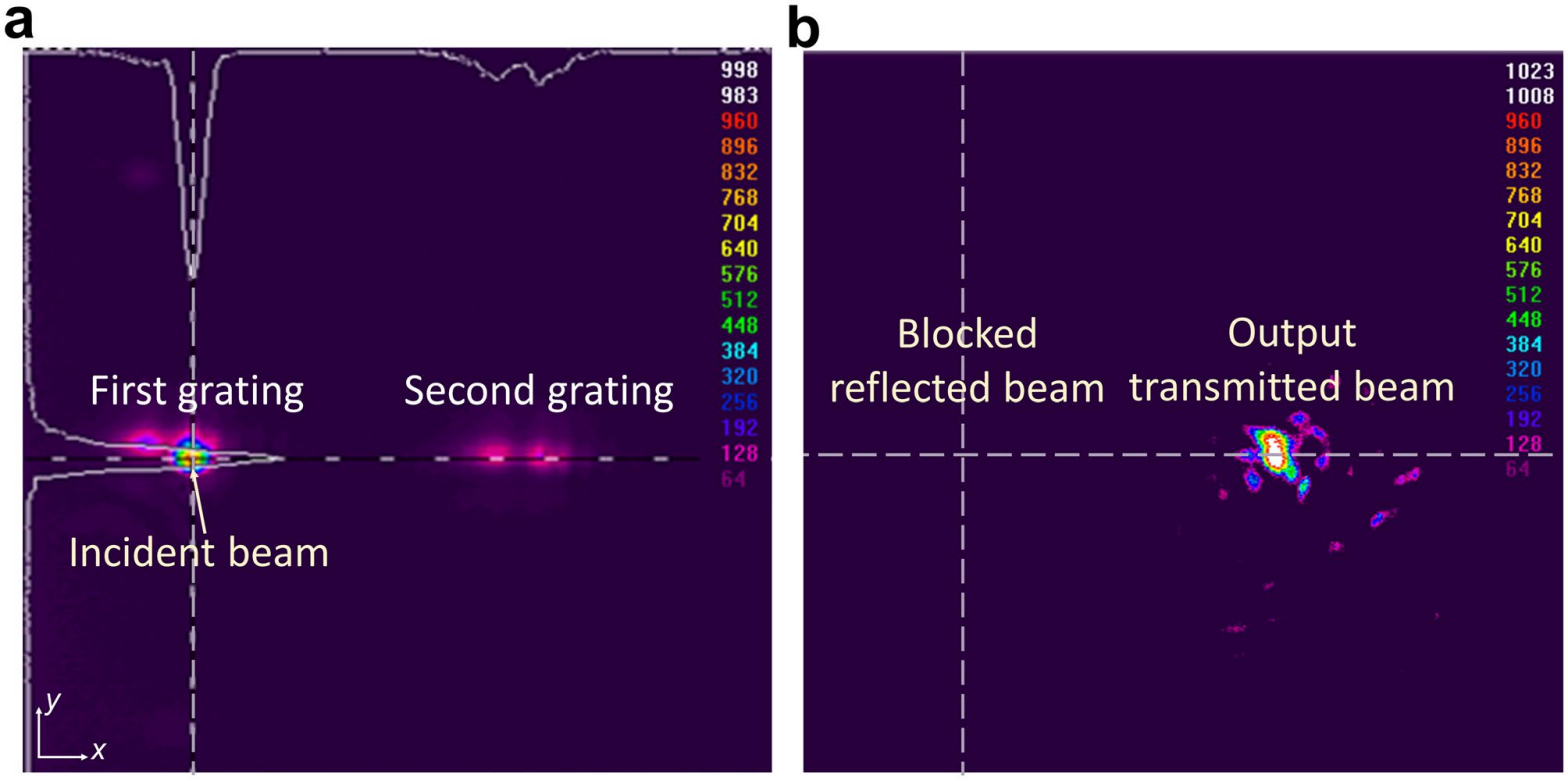
Alignment and measurement procedure. (**a**) Extracted image from the camera frame grabber showing the reflection of the incident beam from a tunable laser, and a projection of the gratings for the air-optimized PSSG structure when the top illuminator is on. (**b**) On-resonance light outcoupled by the second grating, extracted using the frame grabber while blocking the reflected beam from the first grating.

The alignment process ensured the optimal functioning of the experimental setup for precise and reliable data collection. To ensure accurate positioning of the sample, a sample holder arm was connected to a positioner stage equipped with piezo actuators for precise positioning. The initial step involved precisely aligning the optical elements, using a reflective flat gold area to verify that the beam was correctly aligned and focused. The next task was to align the beam to our PSSG sample. This required locating the gratings on the sample and ensuring the light was correctly coupled into one of the PSSGs as the input grating. The visible camera positioned on top of the sample surface facilitated the precise positioning of the incident beam on the grating. When the incident beam interacted with a grating, a spot was visible on the top camera. To further enhance the alignment accuracy, we activated the top illuminator. This illuminator allowed us to capture a projection of the gratings on the IR camera. Using this technique, we could observe both the incident beam spot and the grating projection, confirming that the beam was well-positioned on the input PSSG, as shown in Fig. 5a.

In Fig. 5a, we present an image captured using the IR camera, showcasing the alignment procedure we employed. Once the incident light was effectively coupled to the input PSSG, we proceeded by turning off the top illuminator and using a black metal blocker to obstruct the reflected beam in the light path. The remaining light that emerges from the second PSSG corresponds to the transmission of the SPPs along the top Au surface of the sample, demonstrating successful light coupling between the two gratings, as shown in Fig. 5b. In the following section, we further detail the structure performance and the measurement procedure.

### Measurement results

As previously mentioned, our measurements began with testing the air-optimized PSSG. To ensure the successful excitation of SPPs in the structure and to optimize the alignment between the incident beam spot and the input grating, we initially focused on measuring the reflectance from the input grating. The measurements were taken relative to a reference as a flat gold region devoid of gratings.

We followed the same procedure discussed in the previous section to find a grating and roughly locate the incident beam on the input grating. After that, we used the piezo actuators on the stage positioner to make finer adjustments to the sample position and find its optimized location. After fine-tuning the sample height, adjusting the x-direction for precise centering and coupling the beam to the input grating, we proceeded to accurately adjust the sample position along the y-direction (shown on the axes inset in Fig. 5a). This step is of utmost importance, as avoiding any beam deviation off-grating is critical, ensuring efficient coupling of the PSSG structure.

**Figure 6:**
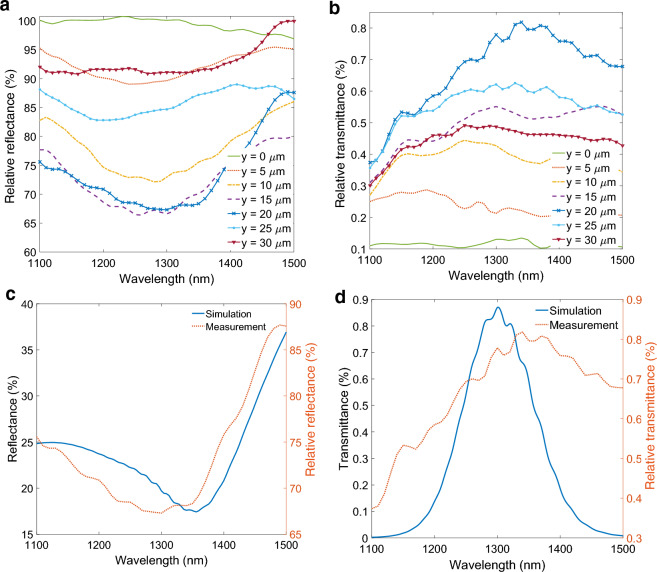
Measured (**a**) reflectance and (**b**) transmittance spectra of the air-optimized PSSG structure at different sample locations. Simulated and measured (**c**) reflectance and (**d**) transmittance spectra of the air-optimized PSSG structure for the best alignment.

In Fig. 6a, the measured reflectance using the supercontinuum source of the air-optimized PSSG structure is presented while varying the sample position. At $y = 20 \mathrm{\mu m}$, the clearest resonance behavior is observed. However, when the sample is positioned far from this optimal location, the beam spot moves off-grating, leading to the disappearance of the resonance in the measured data.

After determining the optimal sample location exhibiting the strongest resonance, we proceeded to block the reflected light and measure the transmittance, representing the light coupled out from the second PSSG. Figure 6b gives the measured transmittance of the air-optimized PSSG for various sample positions. Similar to the reflectance characteristics, a prominent resonance is observed in the transmittance of the structure at the optimal position, confirming the successful excitation of SPPs and their outcoupling by the second grating.

The Fig. 6c,d compare simulation and measurement results for the reflectance and transmittance, respectively. Despite the simplifications in the 2D simulation model and the presence of fabrication imperfections, the overall behavior of the reflectance and transmittance responses in measurements and simulations agree well. Thus, our simplified 2D design approach is useful and efficient to design and optimize PSSGs.

In order to further justify the performance of our PSSG configuration in light-plasmon coupling and prove the efficacy and robustness of the employed experimental setup for sensing applications, we present investigations on the behavior of the water-optimized PSSG structure in the following section.

### Investigation of the water-optimized PSSG structure

**Figure 7:**
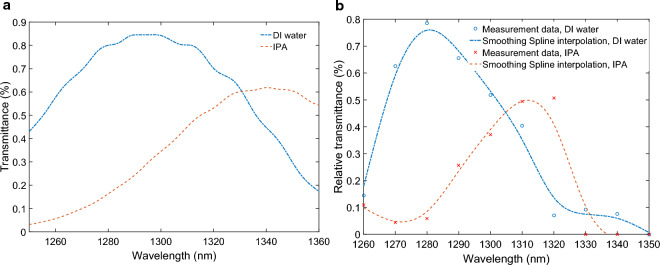
Sensitivity of the water-optimized PSSG structure. (**a**) Simulated and (**b**) measured transmittance spectra as the bounding medium is changed from DI water to IPA.

The Fig. 7a presents the computed transmittance spectra for the water-optimized PSSG structure as the bounding medium is changed from de-ionized (DI) water (refractive index of 1.3223 at ${\lambda }_{0} = 1.31 \mathrm{\mu m}$)^23^ to IPA (refractive index of 1.3750 at ${\lambda }_{0} = 1.31 \mathrm{\mu m}$)^24^. It can be observed that for DI water, the PSSG demonstrates a similar coupling efficiency to the air-optimized structure. Moreover, the change in the bounding medium from DI water to IPA results in an evident shift in the transmittance spectra, indicating a high sensitivity of about 950 nm/RIU. This high sensitivity makes the water-optimized PSSG structure highly promising for sensing and biosensing applications, where small changes in the refractive index can be accurately detected and utilized for various analytical purposes.

To further validate the efficacy of our design and assess its sensitivity experimentally, we obtained measurements with DI water and IPA placed on the sample surface. We emphasize that our design and interrogation setup ensure that the fluids and the optical components are on opposite sides of the substrate. This arrangement prevents any misalignments or disruptions in the optics when exchanging fluids and ensures the stability and reliability of measurements.

The Fig. 7b gives the measurement results for the water-optimized PSSG structure. We utilized a tunable laser capable of providing high-intensity beam spots that are easily detectable with the IR camera. Following the alignment approach described earlier, we precisely located the beam spot on the input PSSG. Subsequently, we blocked the reflected beam to isolate and capture the transmitted beam, corresponding to the light outcoupled by the output PSSG. The same approach was applied to study the case of IPA. Comprehensive details and data showing the captured beams at different wavelengths for DI water and IPA are shown in Supplementary Figures S4 and S5, respectively. The outcoupled beam intensity varies across different wavelengths for DI water and IPA.

To quantify and determine the sensitivity of the water-optimized PSSG structure, we defined a constant region of interest (ROI) encompassing the output beam in each captured image. By extracting the pixel values within the ROI and correlating them to the power measured by the power meter through calibration measurements, we obtained the transmittance spectra for both DI water and IPA, as shown in Fig. 7b. The transmittance spectra show a clear shift in the resonance wavelengths due to the changes in the refractive index of the bounding medium, leading to a sensitivity of approximately 760 nm/RIU, close to the theoretical value. The measured sensitivity compares well to various sensors reported in the literature based on propagating surface plasmons and localized surface plasmon resonances^25–33^. While there exist some plasmonic sensors with higher reported sensitivities^34–37^, the plane of our structure separates the interrogation optics from the fluidics which enables a robust measurement setup well-suited to practical sensing and biosensing applications.

## Conclusion

We investigated theoretically and experimentally plasmonic slit gratings (PSSGs) designed to achieve directional coupling between an incident light beam and surface plasmon polaritons (SPPs) propagating on the surface of the structure. We also investigated mirrored PSSG pairs interconnected by a plasmonic slab waveguide, providing a structure that can be interrogated in transmission, where the input and output interrogation optics are located on one side of the substrate with the fluidics situated on the other side. The structure offers a high sensitivity to changes in the refractive index of the bounding environment (760 nm/RIU, measured) and robustness to fabrication tolerances, ensuring reliability in practical implementations. The structure holds significant promise for applications in sensing.

### Supplementary Information

Supplementary Information.

## Data Availability

All the data generated by this study are included in the manuscript.
