# Supplementary material for: Plasmonic slanted slit gratings for efficient through-substrate light-plasmon coupling and sensing
Source: Sci Rep. 2024 Jan 24;14:2084. doi: 10.1038/s41598-024-52564-9 (PMC11291749; doi:10.1038/s41598-024-52564-9)
Supplement: Supplementary file 1 — Supplementary Information. [file 41598_2024_52564_MOESM1_ESM.pdf]

Supplementary information for

"Engineering plasmonic slanted gratings for efficient light-plasmon coupling and sensing applications"

Fatemeh Fouladi Mahani, Luis Angel Mayoral Astorga, Hyung Woo Choi, Arash Mokhtari, and Pierre Berini

Fabrication Methodology

We employed focused ion beam (FIB) milling using the Zeiss Orion Nanofab to fabricate ordered arrays of slanted gratings. The gallium ion beam source was tilted 46° with respect to the imaging helium-source axis. By default, a substrate was located along the middle of the gun-column at an eccentric position towards the imaging and milling ion sources. Before FIB milling, 30 nm of Cr and 300±10 nm of Au were sequentially deposited on a fused silica substrate by e-beam evaporation at deposition rates of 0.1 A/s and 0.5 A/s, respectively. The deposited Au/Cr bilayers are shown in Fig. S3.

A FIB Ga probe was set at an acceleration voltage of 30 kV and a probe current of 100 pA to synchronize the designed and milled patterns. The milling parameters used to define the slanted gratings are: (i) a dwell time of $1\ \mu s$, (ii) a beam step size of 2 nm, and (iii) a total dose of 1.26 nC/μm^2 for the ion beam exposure. The number of milling patterns was carefully chosen to achieve appropriate areal coverage of the gratings. For the air-optimized PSSG, there are 10 slits, while for the water-optimized PSSG, there are 16 slits, ensuring that the length of the gratings falls within the range of $15 \pm 1\ \mu m$. The width of the PSSGs is $8\ \mu m$.

The milling procedure was performed in two steps, with one step for the input grating and another for the output grating, necessitated by the fixed nature of the ion source. Once the input grating milling was completed, the substrate was rotated 180°, and the same pattern

was aligned away from the input grating to fabricate the output grating. This process resulted in a 40 ± 2 μm distance between the input and output gratings.

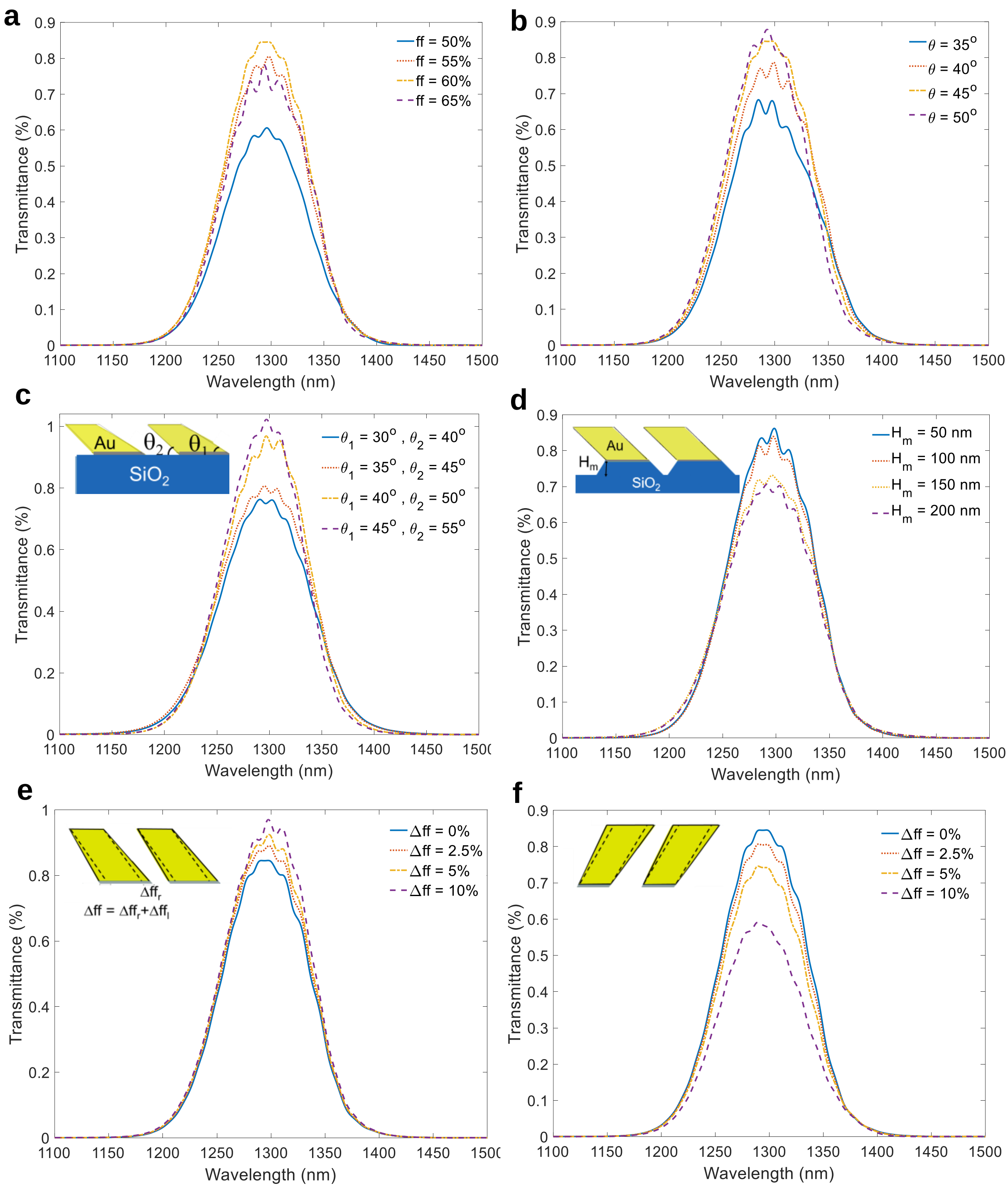

Figure S1 | The effect of variations in the design parameters and fabrication imperfections on the response of the air-optimized PSSG structure. Simulated transmittance of the air-optimized PSSG structure while changing (**a**) the fill factor and (**b**) angle of slants. The effect of (**c**) non-parallel slants and (**d**) milling into the substrate. The effect of (**e**) overcut and (**f**) undercut of slants on the structure performance.

Fig. S1 explores the robustness of the air-optimized PSSG response to different design parameters and fabrication imperfections. In particular, Fig. S1(a) investigates the influence of the fill factor (ff), which denotes the ratio of the grating width to the period, on the transmittance of the structure. This ratio directly affects the effective index of the PSSGs. Consistent with our theoretical design in Table 1, we observe that when ff is set at 60%, the coupling efficiency between the incident light and SPPs reaches its maximum, resulting in higher transmittance levels. However, the structure is robust against slight deviations from the optimal ff, leading to no significant changes in the coupling efficiency.

Fig. S1(b) demonstrates the effect of variations in the slant angle (θ) on the structure response, an essential factor during the FIB milling process. The results indicate that the device behavior also offers stability to minor deviations in the slant angle. Another possible fabrication error is the creation of slanted profiles with non-parallel sidewalls, which have different slant angles on each side, as shown in Fig. S1(c). As observed, non-parllel sidewalls with slight deviations of the slant angle do not have significant effect on the structure response.

Having overcut or undercut slants might also happen during the fabrication, as shown in Fig. S1(e) and (f). Here, the change in the ff, $\Delta\mathrm{ff}$, is defined as the summation of the right and left ff changes, $\Delta\mathrm{ff}_r+,\Delta\mathrm{ff}_l$. Notably, up to 5% change in the ff due to the overcutting and undercutting effects does not change the response much, indicating the design's robustness against fabrication errors.

An additional practical consideration pertains to variations in the depth profile resulting from the angled milling process, potentially leading to the partial removal of the quartz substrate, as illustrated in Fig. S1(d). Even with a partial etch of up to 200 nm, the structure's behavior remains stable. These results show an excellent robustness of the device to fabrication imperfections.

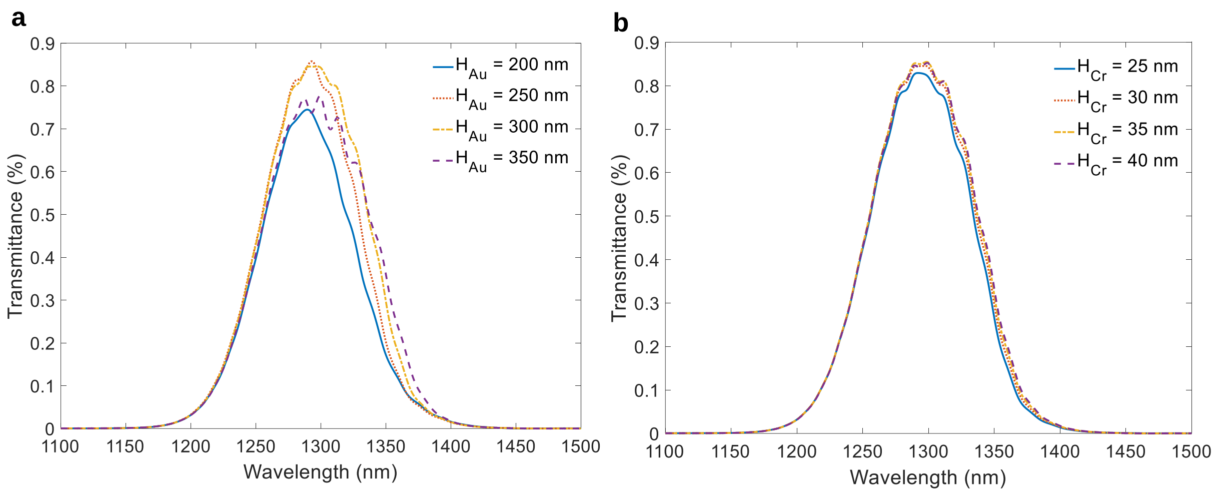

Figure S2 | Effect of Au and Cr thicknesses on the response of the air-optimized PSSG structure. Simulated transmittance of the air-optimized PSSG structure while varying the thickness of (**a**) Au (H_{Au}) and (**b**) Cr (H_{Cr}).

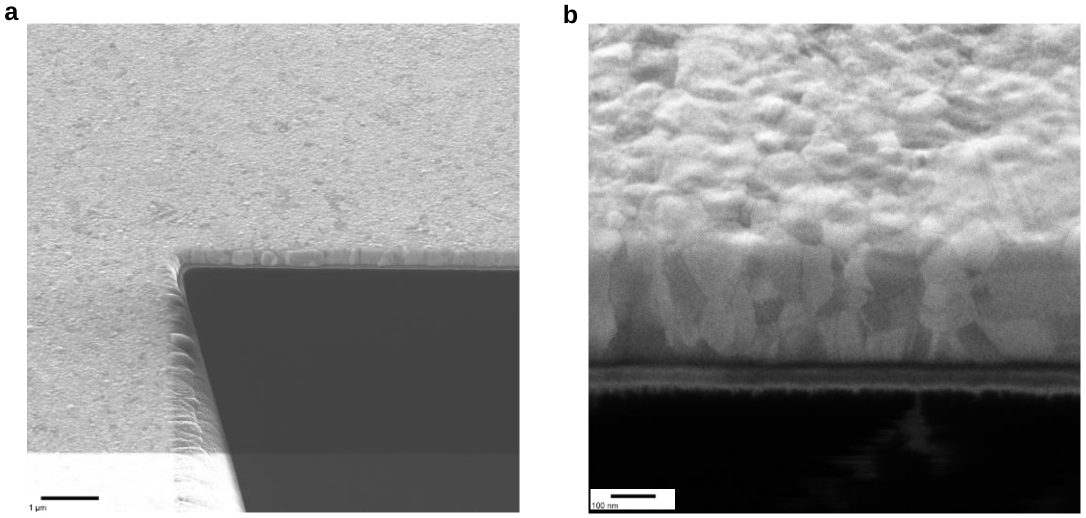

Figure S3 | Analysis of the deposited Au/Cr bilayers. (**a**) Helium ion microscope image of a trench milled with gallium beam in a region devoid of gratings to analyze the Au/Cr layer deposition. (**b**) Helium ion microscope cross-sectional image of the Au/Cr bilayers within the trench shown in (**a**).

The successful deposition of Au/Cr materials is another important aspect to be considered. Fig. S2(a) and (b) illustrate the impact of variations in the Au and Cr thicknesses, respectively, on the structure's behavior. As depicted, the PSSG structure exhibits robustness to small changes in the Au/Cr thicknesses.

Fig. S3 confirms the successful deposition of Au/Cr bilayers. The structure's robustness to slight variations in metal thickness provides confidence in the stability and reliability of our design, even with minor fluctuations in the realized thicknesses. This resilience is a significant

advantage as it ensures the consistent and predictable performance of the PSSG structure in practical applications.

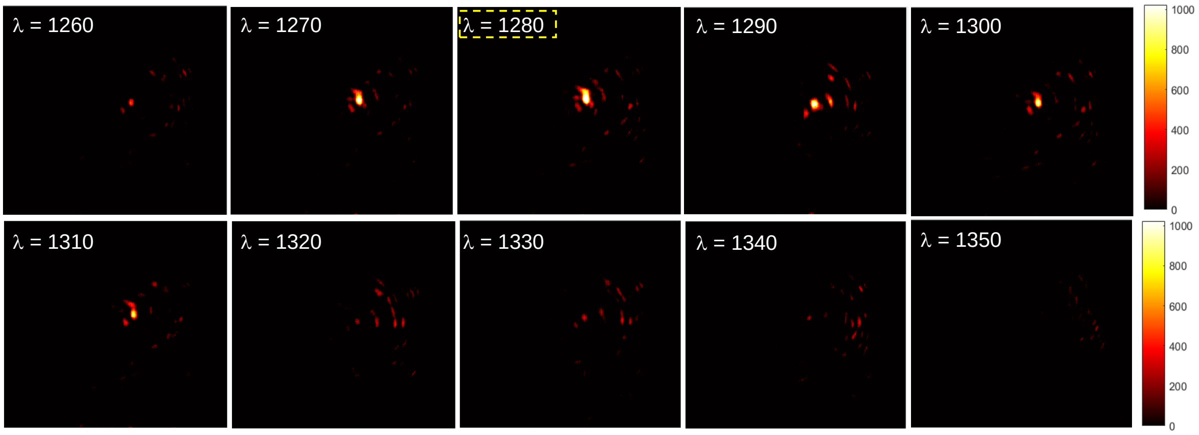

Figure S4 | Light intensities coupled out through the output grating, extracted using a frame grabber card for DI water at different wavelengths.

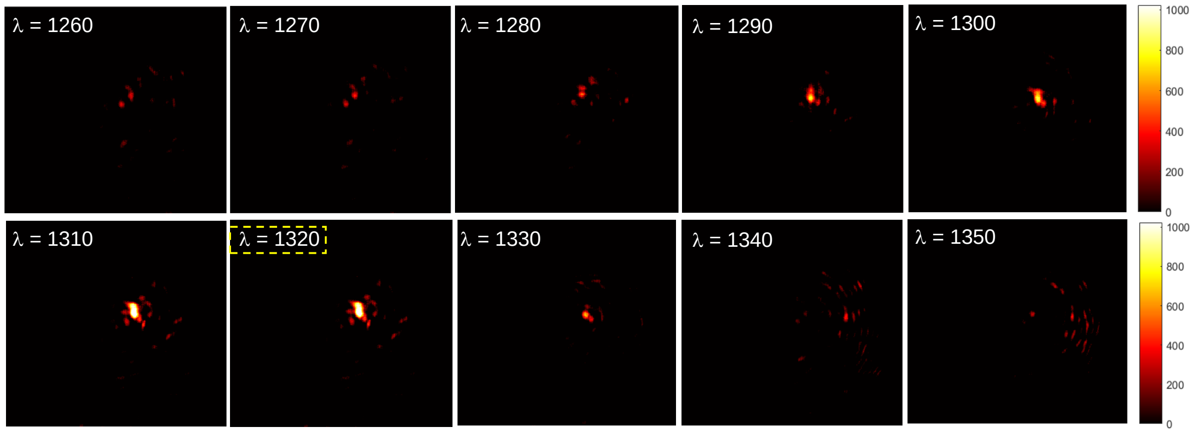

Figure S5 | Light intensities coupled out through the output grating, extracted using a frame grabber card for IPA at different wavelengths.

Figs. S4 and S5 showcase the comprehensive analysis of the outcoupled light intensities at different wavelengths, for DI water and IPA, respectively. Both figures show that the beam intensities exhibit notable variations across different wavelengths. Moreover, the wavelength of the maximum intensified beam shifts for DI water and IPA cases, highlighting the sensitivity of the SPR to the refractive index of the surrounding medium.
